# GPR158, an Orphan Member of G Protein-Coupled Receptor Family C: Glucocorticoid-Stimulated Expression and Novel Nuclear Role

**DOI:** 10.1371/journal.pone.0057843

**Published:** 2013-02-25

**Authors:** Nitin Patel, Tatsuo Itakura, Jose M. Gonzalez, Stephen G. Schwartz, M. Elizabeth Fini

**Affiliations:** 1 Institute for Genetic Medicine, Keck School of Medicine, University of Southern California, Los Angeles, California, United States of America; 2 Doheny Eye Institute, Los Angeles, California, United States of America; 3 Bascom Palmer Eye Institute, University of Miami Miller School of Medicine, Miami, Florida, United States of America; Thomas Jefferson University, United States of America

## Abstract

Members of the large G protein-coupled receptor (GPCR) clan are implicated in many physiological and disease processes, making them important therapeutic drug targets. In the present study, we follow up on results of a pilot study suggesting a functional relationship between glucocorticoid (GC)-induced ocular hypertension and GPR158, one of three orphan members of the GPCR Family C. GC treatment increases levels of GPR158 mRNA and protein through transcriptional mechanisms, in cultured trabecular meshwork (TBM) cells derived from the eye's aqueous outflow pathway. Like treatment with GCs, transient overexpression of GPR158 stimulates cell proliferation, while siRNA knockdown of endogenous GPR158 has the opposite effect. Both endogenous and overexpressed GPR158 show an unusual subcellular localization pattern, being found almost entirely in the nucleus. However, when cells are treated with inhibitors of clathrin-mediated endocytosis, GPR158 is shifted to the plasma membrane. Mutation of a bipartite nuclear localization signal (NLS) in the 8^th^ helix also shifts GPR158 out of the nucleus, but in this case the protein is found in vesicles localized in the cytoplasm. These results suggest that newly synthesized GPR158 first traffics to the plasma membrane, where it rapidly undergoes endocytosis and translocation to the nucleus. Significantly, mutation of the NLS abrogates GPR158-mediated enhancement of cell proliferation, indicating a functional requirement for nuclear localization. GPR158 overexpression upregulates levels of the cell cycle regulator cyclin D1, but mutation of the NLS reverses this. Overexpression of GPR158 enhances the barrier function of a TBM cell monolayer, which is associated with an increase in the levels of tight junction proteins ZO-1 and occludin, similar to reported studies on GC treatment. Regulated paracellular permeability controls aqueous outflow facility *in vivo*. Since GCs stimulate GPR158 expression, the result is consistent with a role for elevation of GPR158 expression in GC-induced ocular hypertension.

## Introduction

The G-protein-coupled receptors (GPCRs) comprise a large superfamily of cell-surface proteins characterized by the presence of a seven transmembrane (7TM) domain [1]. Activated by a broad range of ligands, GPCRs are implicated in many important physiological and disease processes, making them therapeutic targets for a large percentage of current pharmaceuticals [2]. Sequencing of the human genome revealed about 800 previously unknown ‘orphan’ receptors of the GPCR superfamily [3]. The current challenge is to functionally characterize and de-orphanize these receptors, by identification of their endogenous agonists. GPCRs have been classified into seven families based on phylogenetic analysis of the 7TM domain [4]. Human GPCR Family C contains 22 receptor subtypes [5]–[6], including eight metabotropic glutamate receptors (mGlu), and two gamma-aminobutyric acid (GABA) type B receptors (GABBR1 and GABBR2), both with important functions in the central nervous system. Seven of the Family C receptors are orphans, with roles in health and disease largely unknown.

The GPCR Family C orphan GPR158 came to our attention through a small pharmacogenomic study we conducted on risk for glucocorticoid (GC)-induced ocular hypertension (OH) [7]. We found very little information on this gene. Phylogenetically, GPR158 is most closely related to the GABA receptors [5]. Bioinformatics websites such as GeneCards [8] and the Human Protein Atlas (HPA) [9] revealed that GPR158 is expressed at highest levels in the brain, but also in a variety of other cell types. Recently it was shown that the closely related orphan GPR179 is required for depolarizing bipolar cell function in the retina, and is mutated in autosomal-recessive complete congenital stationary night blindness [10]. Most recently, both GPR179 and GPR158 were shown to recruit regulator of G protein signaling (RGS) complexes and thus regulate activity of other GPCRs [11].

In the study reported here, we characterize GPR158 using cells derived from the trabecular meshwork (TBM) tissue of the eye's aqueous outflow pathways, the function of which is affected by GCs. We find an unusual nuclear localization for GPR158, demonstrate mechanisms for nuclear trafficking, and show that this has functional consequences. Like treatment with GC, overexpression of GPR158 enhances cell proliferation as well as the barrier function of a TBM cell monolayer. Regulated paracellular permeability of the TBM barrier controls aqueous outflow facility *in vivo*. Since GCs stimulate GPR158 expression, the results are consistent with a role for elevation of GPR158 expression in GC-induced OH, which can lead to primary open angle glaucoma (POAG).

## Materials and Methods

### Reagents and oligonucleotide primers

The GCs, dexamethasone (Dex) and triamcinolone acetonide (TA), were purchased from Sigma-Aldrich Corp. (St. Louis, MO). The stock solutions of Dex and TA were prepared in ethanol. The transfection reagent, Lipofectamine LTX with PLUS reagent was obtained from Invitrogen (Carlsbad, CA). Horseradish peroxidase-conjugated secondary antibodies were obtained from Santa Cruz Biotechnology (Santa Cruz, CA). All other reagents were purchased from Sigma. Concanavalin A (ConA) and chlorpromazine (CPZ) were purchased from MP Biomedicals LLC (Solon, OH). High-Fidelity DNA polymerase, Phusion was procured from Finnzymes Inc. (Woburn, MA). Two GPR158 antibodies: anti-C-terminal (epitope AA 914-1052) and anti-N-terminal (epitope AA 24-74), were purchased from Sigma-Aldrich Corp. (St. Louis, MO). Green fluorescent protein (GFP) and cyclin D1 antibodies were procured from Rockland Immunochemicals (Gilbertsville, PA). ZO-1 and occludin antibodies were purchased from Abcam, Inc. (Cambridge, MA). Oligonucleotides primers used for PCR amplification were obtained from Integrated DNA Technologies (San Diego, CA). **Table 1** provides the sequences of all oligonucleotides used as primers in this study. The primers designed to detect GPR158 and β-actin are very specific as confirmed by the UCSC genome browser In-Silico PCR, and the amplified product was also confirmed by sequencing for specific detection of GPR158 in the USC DNA sequencing core facility.

**Table 1:** Oligonucleotide primers used in this study.

Gene/Fragment	Method	Forward sequence	Reverse sequence
GPR158 (FraA)	PCR	ggccgctagcatgggagccatggcttaccc	tatagggcccctgattggggc
GPR158 (FraB)	PCR	tatagggccccggggcctgggcc	ggccgagctcattgtgaactgcaac agcc
GPR158 (FraC)	PCR	gcatatgctagcgagctcatcatc tctgctatattc	gcaggctctagactacactttaaaactatc cca
GPR158 promoter (-1053/+25bp)	PCR	ggccggtaccctcccttctccctcttttc	ggccgctagcgtaaggggtaagccatgg
NLS-M1	SDM	gacattcgggacgagctgcaacaactctatgcccaactg	cagttgggcatagagttgttgcagctcgt cccgaatgtc
NLS-M2	SDM	ctggaaatatataaaagacagcagatgatcacaaacaacccccac	gtgggggttgtttgtgatcatctgctgtcttttatatatttccag
GPR158	RT-PCR	atattgctacagaagcatatgag	atatttccagttgggcatagag
Myocilin	RT-PCR	gccagtttttgagtatgacct	gtttgttcgagttccagattc
Aquaporin-1	RT-PCR	tgccatcggcctctctgta	cagggttaatcccacagcca
β-actin	RT-PCR	cattgccgacaggatgcaga	ctgatccacatctgctggaa
GAPDH	RT-PCR	aacctgccaagtacgatgacatc	gtagcccaggatgcccttga

The abbreviations are: GPR158, G protein-coupled receptor 158; Fra, fragment; GAPDH, glyceraldehyde 3-phosphate dehydrogenase; PCR, polymerase chain reaction; RT-PCR, reverse transcriptase–polymerase chain reaction; SDM, site-directed mutagenesis.

For knockdown experiments, we used a pool of three custom designed siRNA oligonucleotides (GenePharma Co. Ltd, Shanghai, China). The specificity of the sequences for the target gene GPR158 was confirmed by BLAST search. The synthesized siRNAs were purified by HPLC, then 2′-O-methyl modified at position 2 to deactivate off-target activity without any compromise in silencing effect. A scrambled control siRNA sequence was also developed by GenePharma, which contained the same GC% as the GPR158 siRNAs but was not homologous to any known eukaryotic RNA sequence. Transfection of cells with the GPR158 siRNA pool decreased the endogenous GPR158 protein level in a concentration dependent manner, with an 80-90% knockdown of GPR158 protein at 100 nM, a result consistently seen across many different experiments (Figure S1). In contrast, transfection with the GPR158 siRNA reagent had no effect on actin protein levels. Moreover, the control scrambled siRNA reagent had no effect on either GPR158 or actin protein level at the same concentration (Figure S1).

### Cells and tissues

An immortalized human TBM cell line, TM-1, was generously donated by Dr. Donna Peters, University of Wisconsin-Madison [12]. We refer to this cell line as ‘TBM-1’ throughout this paper, so as not to confuse it with the GPCR 7TM domain. Cells were grown in low-glucose Dulbecco's modified Eagle's medium (DMEM) containing 10% FBS (Atlanta Biologicals, Inc., Norcross, GA), 2 mM L-glutamine, 2.5 µg/mL amphotericin B, and 25 µg/mL gentamicin, as previously described [12].

Primary TBM cells were isolated from fresh corneal rims remaining after removal of the cornea for surgical transplantation from healthy human donor eyes. The cells were isolated and cultured according to published procedures [12]. Briefly, the TBM tissue was carefully dissected from the rim under the light microscope and transferred in a 48 well-plate containing growth media. The tissue was covered with cover slip and the primary cells were recovered at the bottom of the well. For experimental purpose, the primary cells were confirmed by morphology, by the TBM cell marker aquaporin, and by the induction of the TBM cell marker myocilin by Dex treatment for 10 days. For experiments, cells were maintained in reduced serum (2.5%) for indicated time periods. The control cells were treated with the same dilution of ethanol alone. Unless otherwise mentioned, the cells were treated with 250 nM concentrations of both Dex and TA.

Prostate cancer PC-3 cells were a kind gift of Dr. Gerhard Coetzee (Keck School of Medicine of USC). These cells were used in some experiments because they express GPR158 at higher constitute levels that TBM cells. The mouse prostate (which also expresses higher constitutive levels of GPR158 than the TBM) was obtained from a 10.5-months old mouse with a mixed genetic background of C57B/6XDBA2, 129/BALB/c, and FVB/N. All animal experiments were in accordance with institutional guidelines and were approved by the Institutional Animal Care and Use Committee of University of Southern California. Mice were sacrificed by CO_2_ asphyxiation.

### Quantitative real-time reverse transcription-PCR (qRT-PCR)

Total RNA was isolated using Aurum total RNA mini kit (Bio-Rad, Hercules, CA) and qRT-PCR analysis was carried out using iScript one-step RT-PCR kit with SYBR Green (Bio-Rad) on an ABI PRISM 7900 HT sequence detection system (Applied Biosystems, Foster City, CA), according to the manufacturer's instructions. Relative quantification values of GPR158 mRNA expression were calculated as 2^–ΔΔCt^ by the comparative Ct method, where ΔΔCt =  (Ct target mRNA of treated sample- Ct reference gene of treated sample) - (Ct target mRNA of control sample-Ct reference gene of control sample).

### Western blot analysis

Cytosolic extracts were prepared from untreated and treated (Dex and TA) cells. Briefly, cells were suspended in 200 µL of radioimmunoprecipitation assay buffer containing Tris 50 mM, NaCl 150 mM, SDS 0.1%, sodium deoxycholate 0.5%, NP-40 1% and protease inhibitors cocktail (RIPA lysis buffer) and after 20 min cells were centrifuged at 10,000 g for 10 min and supernatants were collected. For the preparation of mouse prostate lysates, the tissue was pulverized and then lysed in RIPA buffer and the same procedure was followed for the isolation of lysates as above. In separate experiments, nuclei were isolated from cells after incubation in hypotonic salt buffer according to the standard procedure. For peptide N-glycosidase F (PNGase F) experiment, 30 µg of cell lysates were treated with 500 units of PNGase F for 3 hrs at 37°C. Proteins in the extracts were separated by SDS-PAGE and transferred to PVDF membranes. Membranes were probed with GPR158 antibody and were developed by chemiluminescence with reagent Lumigen TMA-6 (GE Healthcare UK limited, Buckinghumshire, UK) and images were captured with Fujifilm imaging system (LAS-4000; Fujifilm, Tokyo, Japan). Protein loading was monitored by stripping and reprobing of the membrane with β-actin antibody.

### Generation of GPR158 promoter luciferase construct

The 1.078 kb segment spanning the region between -1053 to +25 bp, relative to the transcription start site of GPR158 mRNA was PCR-amplified using the forward primer containing KpnI restriction enzyme site and reverse primer containing NheI site, as listed in Table 1, according to the standard procedure utilizing Phusion high fidelity DNA polymerase. BAC clone RP11-80K21 was used as a template (Empire Genomics, Buffalo, NY). Briefly, PCR was performed using 100 ng of BAC clone DNA using conditions: 95°C for 30 sec, 60°C for 60 sec, and 72°C for 60 sec for 30 cycles. PCR product was cloned into pGL3 vector (Promega, Madison, WI) and DNA sequences were confirmed by sequencing (Microchemical Core Facility, USC Norris Comprehensive Cancer Center).

### Transient transfection and reporter assay

TBM-1 cells were transfected with Lipofectamine LTX reagent as per supplier's instructions. Briefly, the cells were seeded in six-well plates at a density of 1–2×10^5^ cells/well in 2 mL complete medium and grown for 1–3 days until 70–80% confluence. DNA (2 µug) was diluted in 500 µL Opti-MEM I, PLUS reagent (2 µL) was added and incubated for 5 min. Lipofectamine LTX (4.5 µL) was added, and the complexes were formed by incubation for 30 min. Cell medium was replaced with fresh 2 mL antibiotics free medium and the mixture was added, and incubated for 6 hrs. Following incubation, the complexes were replaced with complete growth medium. Post-transfection, the cells were kept in complete medium overnight and then treated with Dex or TA for the indicated time. The cells were lysed and analyzed for luciferase activity using VICTOR3V plate reader (PerkinElmer, Shelton, CT) and beta-galactosidase activity using kits (Promega, Madison, WI). Luciferase values were normalized to beta-galactosidase values. Data are expressed relative to the activity of the promoter-less pGL3 basic vector.

### Cloning and overexpression of GPR158

The size of GPR158 gene is large (3.648 kb). In order to avoid any random mutations resulting from polymerase errors, GPR158 was cloned in three smaller fragments (Fra) including FraA (729 bp), FraB (1129 bp) and FraC (1790 bp) using cDNA isolated from PC-3 cells. Using appropriate forward and reverse primers as indicated in Table 1, all three fragments were PCR amplified, cloned into pGEM-T Easy vector and confirmed by DNA sequencing. For the construction of a full-length gene, all three fragments were isolated from their respective pGEM-T Easy vector using appropriate restriction enzymes and purified using DNA columns (Qiagen Inc., Valencia, CA). The purified fragments were then ligated using DNA ligase to obtain the full-length GPR158 gene. The full-length GPR158 was subsequently subcloned into two mammalian expression vectors: pcDNA 3.1(+) using AflII and XbaI restriction enzymes, and pEGFP-C1 vector with GFP tag at the C-terminus, using NheI and AgeI enzymes.

### Mutagenesis of the bipartite nuclear localization signal (NLS) in GPR158

In order to demonstrate the functional importance of a putative bipartite NLS in nuclear localization of GPR158, we generated three different NLS mutants where lysines are replaced with glutamine. These are designated as NLS-M1 (K^719, 720^Q), NLS-M2 (K^731, 732^Q) and a double mutant NLS-M1+2 (K^719, 720, 731, 732^Q). The specific lysines within the putative bipartite NLS were substituted using the QuikChange site-directed mutagenesis kit (Agilent Technologies, Santa Clara, CA) according to the suppliers' instructions and verified by DNA sequencing (Genewiz Inc, San Diego, CA). Briefly, two complementary mutant primers (125 ng each) containing the desired mutation and 100 ng of template (wild type GPR158-GFP plasmid) in 1× reaction buffer were denatured at 95°C for 30 s followed by 16 cycles of DNA synthesis using Pfu Turbo polymerase using 95°C/30 s, 55°C/1 min, and 68°C/7 min as the cycling conditions. The methylated template was removed by incubation with 10 units of DpnI at 37°C for 1 h. The mutant primer sequences used are listed in Table 1. The double mutant NLS-M1+2 (K^719, 720, 731, 732^Q) was generated using NLS-M1 as a template.

### Immunolocalization

Following treatment with Dex or ethanol, cells were fixed in methanol for 10 min, washed three times in PBS and blocked with 10% horse serum for 45 min. The cells were then incubated with the polyclonal rabbit anti-GPR158 antibody (1∶50 dilution) for 1 hour at room temperature (RT). Cells were subsequently washed with PBS three times and incubated with secondary AlexaFluor 488 rabbit antibodies at 1∶1000 dilution (Invitrogen-Molecular Probes, Eugene, OR) for 1 hour at room temperature. Cells were washed again three times with PBS and mounted with mounting medium containing propidium iodide to visualize nuclei (Vector Laboratories, Burlingame, CA). For ConA treatment, the cells overexpressing either GPR158-GFP or GFP were treated with ConA (0.25 mg/mL) in culture media for 8 hrs. The cells were washed twice with PBS, fixed with paraformaldehyde (PFA) for 20 mins at RT, washed again and mounted with medium containing DAPI. For visualization of NLS mutants of GPR158-GFP fusion proteins, the cells were first fixed with PFA and then mounted with DAPI medium. The slides were imaged using a Nikon Eclipse Ti-E fluorescence microscope and confocal microscope at fixed acquisition parameters.

### Barrier function assay

We examined the effects of GPR158 overexpression on barrier function of a cultured TBM cell monolayer using the *In Vitro* Vascular Permeability Assay (IVP) (EMD Millipore Corporation, Billerica, MA), which measures paracellular permeability. The assay was performed as described earlier [13]–[14]. Briefly, primary human TBM cells were seeded on collagen inserts (20,000 cells/insert). When cells reached 80-90% confluence, they were transfected with either empty vector or GPR158 expression vector using lipofectamine 2000 reagent. The cells were used for the permeability experiment 96 hrs after transfection. In some wells, IL-1alpha (10 ng/ml) or TGF-beta2 (10 ng/ml) was added for 24 hrs prior to assessing permeability, as a positive and negative control, respectively. 100 µl of culture medium containing 1∶40 FITC-Dextran was added in the top insert and the cells were incubated 20 mins at RT. Permeability was determined by measuring the fluorescence of 100 µl of solution from the receiver tray using an excitation/emission wavelength at 485 nm/530 nm with the VICTOR3V instrument. The fluorescence units recorded in untreated or vector transfected cells was set at a value of 1 and the relative permeability was calculated for the treated samples.

## Results

### *In silico* analysis of GPR158 protein

**Figure 1:**
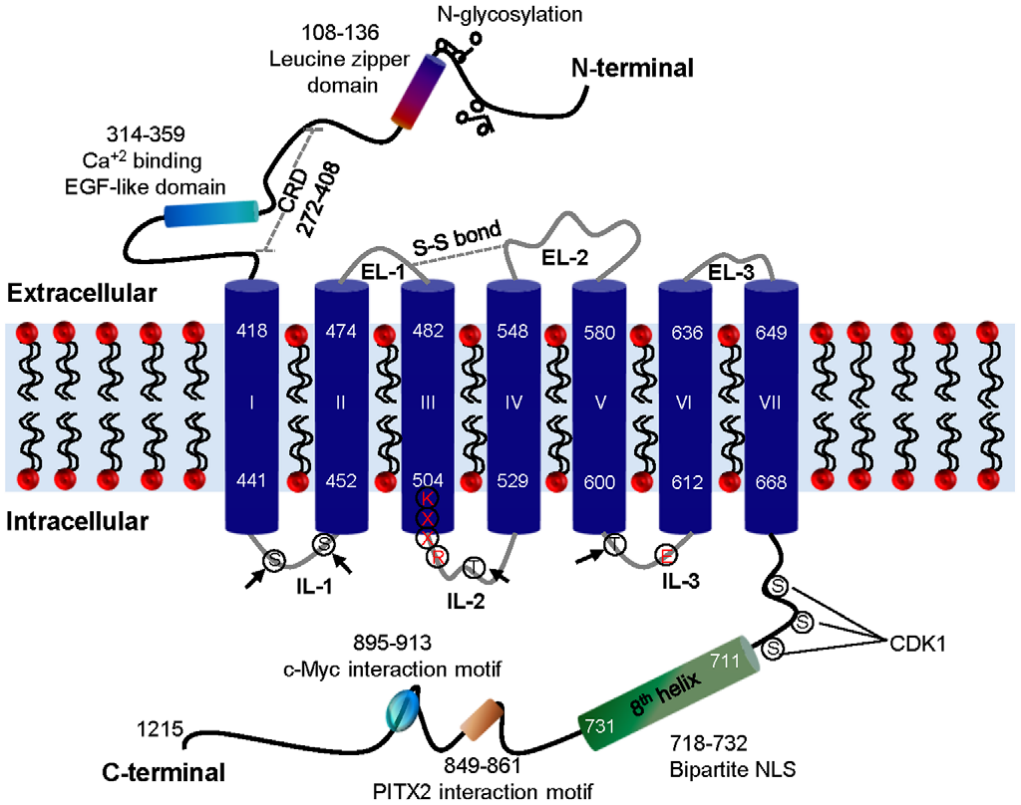
The schematic representation of two-dimensional structure of GPR158. Three extracellular loops (ELs) and three intracellular loops (ILs) connect the seven TM (numbered I-VII). The arrow indicates putative PKC and PKA phosphorylation sites in the ILs. The cysteine residues in EL-1 and EL-2 involved in disulfide (S-S) bond formation are shown as a dotted grey double line. The eighth helix, bipartite NLS, c-Myc and PITX2 interaction motifs, and putative phosphorylation sites for kinases, such as CDK1, are indicated in the C-terminal tail. The leucine zipper domain, EGF like domain, N-glycosylation sites and putative CRD are shown in the N-terminal of GPR158. The conserved amino acid residues, KXXR and E, involved in G protein activation in class C GPCRs are marked in red color. CDK1, cyclin-dependent kinase 1; CRD, cysteine rich domain; EGF, epidermal growth factor; EL, extracellular loop; IL, intracellular loop; NLS, nuclear localization signal; PITX2, paired-like homeodomain transcription factor 2.

GPR158 is predicted to have a protein molecular mass of 135 kDa, as deduced from the cDNA sequence. Results of our *in silico* analysis of the predicted GPR158 protein are depicted in Figure 1. Application of the web-based PSIPRED program for protein secondary structure [15] predicts the characteristic 7TM domain of a GPCR as well as an 8^th^ helix at the proximal end of GPR158's C-terminal cytoplasmic tail (AA 711-731). Use of the sequence pattern and motif search on the EXPASY proteomics server (Swiss Institute of Bioinformatics) revealed the presence of a signal peptide (AA 1-23), Ca^+2^-binding EGF-like domain (AA 314-359) and a leucine zipper domain (AA 108-136) within the N-terminal extracellular domain, and a signature motif characteristic of the metabotropic glutamate receptor family (AA 444-466) at the start of the 7^th^ helix. GPR158 contains several potential N-glycosylation sites, all of them located in the N-terminal domain, but no O-glycosylation sites (NetNGlyc 1.0 and NetOGlyc 3.1 server, Center for Biological Sequence Analysis, Technical University of Denmark DTU). Most Family C GPCRs contain an N-terminal Venus Fly Trap (VFT) domain that is linked to the 7TM domain via the cysteine-rich domain (CRD) and plays an important role in ligand recognition [6]. While GPPR158 lacks the VFT domain [16], we identified eleven cysteine residues near the extracellular domain's distal end, which could form a similar rigid stem structure like the CRD. In addition, GPR158 features the presence of cysteine residues in the analogous locations in EL1 and EL2 as in many GPCRs, involved in a disulfide bond formation, which is thought to be important for ligand recognition [17], [18].

Use of the PredictNLS program on the NucPred server [19] identified a bipartite nuclear localization signal (NLS) motif composed of two basic amino acid stretches, LKKLY and KRKK within the 8^th^ helix present in the GPR158 C-terminal cytoplasmic tail. The NetPhosK 1.0 server, identified several sites in the C-terminal cytoplasmic tail, located between the 7^th^ and 8^th^ helices of GPR158, with the potential to be phosphorylated by protein kinases involved in cell proliferation. These include Ribosomal S6 Kinase (RSK), Cyclin-Dependent Kinase 1 (CDK1), Casein Kinase 1 and Casein Kinase II (CKI and CKII) and DNA-Dependent Protein Kinase (DNA-PK). The UniProt database uncovered experimental evidence for binding of two transcription factors implicated in regulation of cell cycle progression to specific sequences located within the cytoplasmic tail of GPR158, just distal to the 8^th^ helix. The interaction between transcription factor c-Myc and GPR158 was shown in lysates from a human fibroblast cell line through new tandem affinity purification [20] while interaction with PITX2 was identified through a high-throughput mRNA display screen using a human brain cDNA library [21].

### GPR158 expression

**Figure 2:**
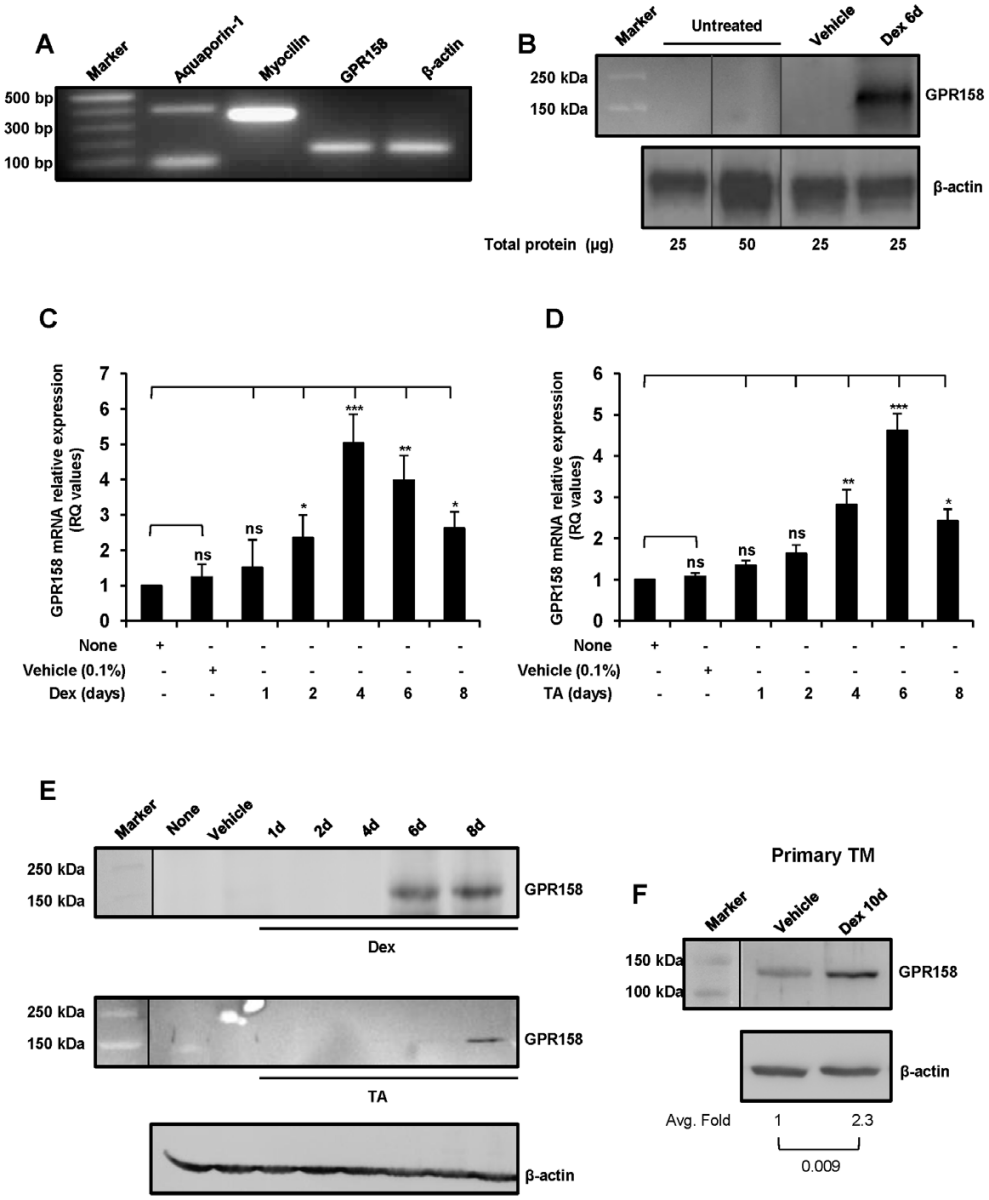
Expression and GC-mediated induction of GPR158 in trabecular meshwork cells. (**A**) RT-PCR analysis of GPR158, aquaporin-1, myocilin and β-actin mRNA expression in TBM-1 cells. (**B**) Western blotting for GPR158 in cellular extracts from untreated and Dex treated (250 nM) for 6 days using anti-C-terminal GPR158 antibodies (1∶1000 dilution). The data are representative of three independent experiments. The vertical line indicates repositioned gel lanes. (**C and D**) TBM-1 cells were stimulated with either Dex (**C**) or TA (**D**) for the indicated time periods. Total RNA was isolated for quantitation of GPR158 mRNA by qRT-PCR. GAPDH was used as a reference gene. The cells treated with ethanol (0.1%) were used as a negative control. The data are representative of three independent experiments. ***P<.001; **P<.01, *P<.05; ns, P>.05. (**E**) The stimulation of TBM-1 cells was carried out with either Dex **(top panel)** or TA **(middle panel)** OR primary TBM cells were treated with either Dex or vehicle alone (**F**), for indicated time points. Following treatment, the cells were lysed with RIPA lysis buffer and cellular extracts were prepared. The protein lysates were then loaded onto SDS-PAGE to perform western blotting for GPR158 protein using indicated anti-C-terminal GPR158 antibodies. The same membrane was stripped and reprobed for β-actin. The data are representative of three independent experiments. (**F**) Quantification of GPR158 protein band intensities was measured by NIH Image J, normalized by β-actin band intensities and expressed in terms of fold expression relative to levels in vehicle treated cells arbitrarily set at 1.0. The statistical analysis was carried out using unpaired Student's t tests (P<0.05). The average fold and P values obtained from three different experiments are indicated in the figure. The vertical line in Fig. 2, E and F indicates repositioned gel lanes.

Expression of GPR158 mRNA and protein was examined in TBM-1 cell cultures (Fig. 2, A and B). Using specific primers listed in Table 1, TBM-1expression of TBM cell-specific markers for aquaporin-1 (Fig. 2A, lane 2) and myocilin (Fig. 2A, lane 3) was confirmed. In addition, we identified the expression of GPR158 mRNA (Fig. 2A, lane 4), however GPR158 protein expression was not detected, despite loading 50 µg of total cell lysates (Fig. 2B, lane 3). Many previous studies have shown that the TBM marker myocilin is undetectable in untreated TBM cells, but expression attains detectable levels when cells are treated with Dex [22]. When we treated TBM-1 cells with 250 nM of Dex for 6 days, GPR158 protein was easily detected (Fig. 2B, lane 4 and 5) as a specific protein band of approximately 150 kDa. This is larger than the predicted size of 135 kDa, however it is consistent with the size recently reported for human GPR158 present in lysates from retina and other tissues [11]. This larger size band could be due to post-translational N-glycosylation, as predicted by NetNGlyc 1.0 server. To test this idea, we treated PC-3 cell lysates with PNGase F to remove N-linked glycans prior to SDS-PAGE analysis (data not shown). This resulted in a shift in molecular mass of GPR158 from 150 to 135 kDa, corresponding to the predicted molecular weight from the deduced amino acid sequence. These results confirm that the identity of the 150-kDa band as N-glycosylated GPR158.

### Stimulation of GPR158 expression by treatment with glucocorticoids

We compared the time course of GC-stimulated GPR158 mRNA expression in TBM-1 cells. We chose the concentration and duration of treatment with Dex or TA based on our dose-response analysis in the GPR158 promoter reporter activity assays as described below. The treatment with Dex (Fig. 2C) or TA (Fig. 2D) resulted in a rapid 4-5-fold increase in GPR158 mRNA, with a peak at 4 and 6 days, respectively. These results were in comparison with 0.1% ethanol vehicle-treated cells (Fig. 2, C and D). Western blotting was used to correlate the stimulation in GPR158 mRNA levels with protein expression in TBM-1 cells. As shown in Fig. 2E, Dex treatment led to increased GPR158 protein levels at both 6 and 8 day time points (top panel), while TA treatment showed increased expression only at 8 days in TBM-1 cells (middle panel). We also examined Dex stimulation of GPR158 protein expression in primary TBM cell cultures. As indicated in Fig. 2F, a specific band was detected in vehicle treated cells of slightly less than 150 kDa. The slight difference in size from that seen in cell lines (described above) is likely due to differential post-translational modifications. The amount of GPR158 protein was approximately 2.3-fold greater when cells were stimulated with Dex treatment for 10 days (Fig. 2F). The choice to stimulate primary cells for 10 days was made based on the published time-course of induction of myocilin with Dex treatment [22]. These results confirm that GPR158 is GC-inducible in both primary and transformed TBM cells.

### Transcriptional regulation of GPR158 by glucocorticoids

**Figure 3:**
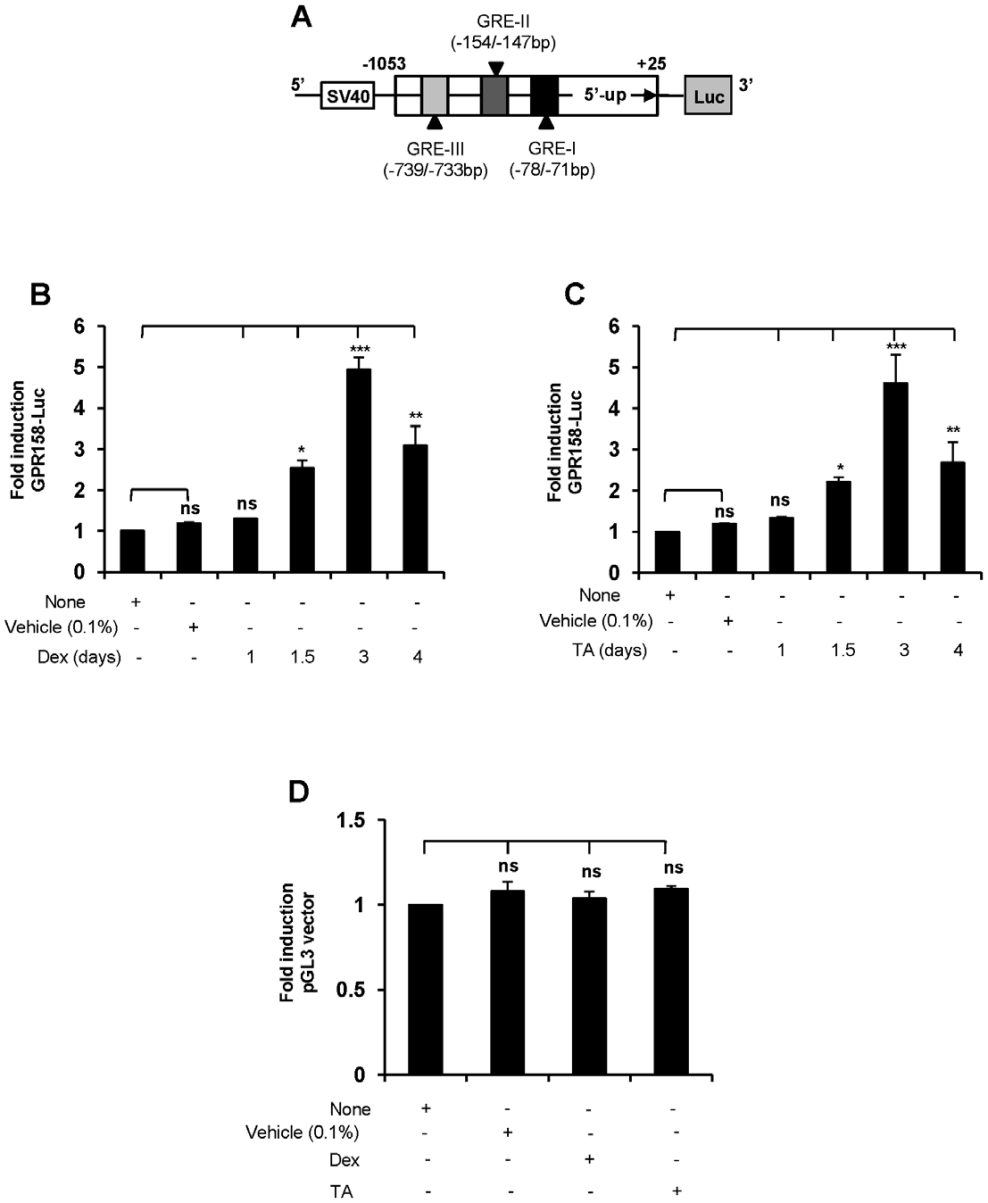
GPR158 promoter activity in TBM-1 cells following GC treatment. (**A**) Schematic illustration of GPR158 promoter (−1053/+25 bp, from transcription start site) indicating the location of three GREs. (**B and C**) TBM-1 cells were cotransfected with GPR158 promoter construct and β-galactosidase plasmid using Lipofectamine LTX reagent, followed by Dex (**B**) or TA (**C**) treatment for indicated time points. (**D**) TBM-1 cells were cotransfected with promoter-less pGL3 vector and β-galactosidase plasmid. Post-transfection, the cells were either left untreated or treated with vehicle control, or Dex or TA. Treatment was for 3 days for TBM-1 cells. (**B, C, and D**) The luciferase activity was normalized to that of the promoterless pGL3 basic vector. Data are expressed as mean ± SEM of three independent experiments ***P<.001; **P<.01; *P<.05; ns, P>.05.

Since Dex and TA stimulated GPR158 mRNA expression in TBM-1 cells, we analyzed the 5′-upstream promoter sequence of GPR158 for the presence of GC responsive elements (GREs). Examination of the sequence (−1053/+25 bp) upstream of the GPR158 transcript revealed the presence of a TATA box at −54 bp region from the transcription start site. Additionally, there are three potential GREs located at different positions that are indicated in schematics Fig. 3A. In order to study the GC-mediated regulation of the GPR158 promoter, we made a luciferase fusion construct containing -1053 bp to +25 bp of GPR158, inserted into the pGL3 basic luciferase reporter vector as described in ‘Materials and Methods’. We carried out a Dex and TA dose (50-1000 nM) response analysis on GPR158 promoter stimulation in TBM-1 cells. Both Dex and TA showed the optimal increase in reporter activity at 250 nM (data not shown). Next, we analyzed the kinetics of the GPR158 transcriptional promoter response to Dex or TA. As shown in Fig. 3B, transfection of GPR158 promoter luciferase plasmid into TBM-1 cells, followed by treatment with Dex resulted in a time dependent increase in reporter activity with the maximal 5-fold increase at 3 days, compared with cells treated with vehicle control. Similarly, TA treatment of cells transfected with GPR158 promoter construct led to increased luciferase activity by 4-fold at 3 days, followed by reduction in activity at 4 days (Fig. 3C). The reporter activity of the promoter-less pGL3 construct did not change by stimulation with either vehicle or Dex or TA for 3 days in TBM-1 cells (Fig. 3D). Therefore, these results indicate that both Dex and TA drive GPR158 promoter activity.

### GPR158 promotes cell proliferation

**Figure 4:**
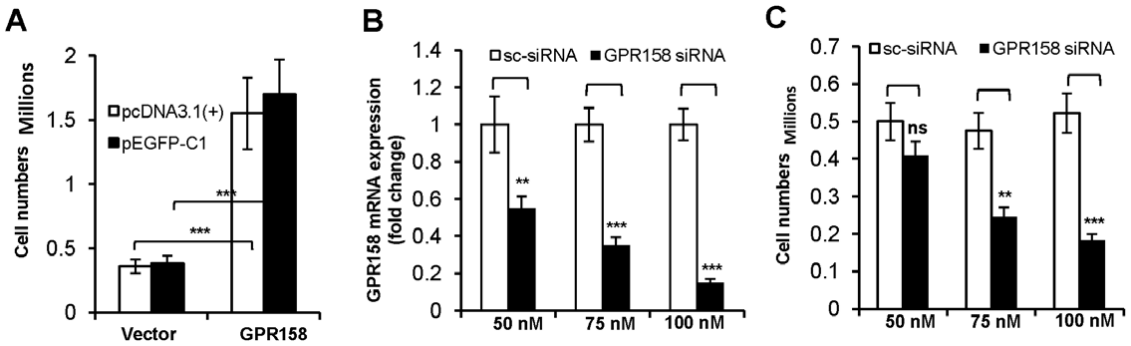
GPR158 regulates the proliferation of TBM-1 cells. TBM-1 cells were transfected at 70–80% confluence using Lipofectamine LTX reagent with either GPR158 expression plasmids or vector alone (**A**) OR either GPR158 siRNA or control scrambled siRNA as indicated (**B and C**) and incubated in growth medium for 3 days in a 6-well culture dishes. (**A and C**) After 3 days of transfection, the cells were trypsinized and counted using trypan blue dye in a hemocytometer chamber for the cells transfected with indicated plasmids. (**B**) Total RNA was isolated for analyzing the levels of GPR158 mRNA by qRT-PCR. β-actin was used as a reference gene. (**A, B and C**) The data represent mean ± SEM of three independent experiments. ***P<.001; **P<.01; *P<.05; ns, P>.05.

To determine the effects of GPR158 on cell physiology, TBM-1 cells were transiently transfected with two different GPR158 overexpression constructs: GPR158-pcDNA 3.1(+) and GPR158- GFP. Representative results are shown in Fig. 4A. The cell density was noticeably higher after three days in GPR158-transfected cultures as compared to control cultures transfected with empty vector (data not shown). When assessed quantitatively, a 4.3-fold and 4.4-fold increase in cell numbers were observed in TBM-1 cells overexpressing GPR158-pcDNA 3.1(+) and GPR158-GFP constructs, respectively, in comparison with their corresponding control vector (Fig. 4A). The increased rate of cell proliferation gradually diminished and returned to normal at 4, 6 and 8 days post-transfection, paralleling the loss of GPR158 protein expression due to loss of transiently transfected DNA, as assessed by western blotting (data not shown).

Performing the converse experiment, we evaluated the effect on TBM-1 cell proliferation of GPR158 down regulation through an siRNA approach. Transfection with a mixture of three different GPR158 siRNAs caused a concentration-dependent down-regulation of endogenous GPR158 mRNA expression with up to an 85% reduction at 100 nM, as estimated using qRT-PCR (Fig. 4B). A parallel reduction in TBM-1 cell proliferation occurred, with the greatest reduction (65%) occurring at 100 nM of GPR158 siRNA (Fig. 4C). In contrast, TBM-1 cells transfected with control siRNA continued to proliferate without an apparent reduction in rate (Fig. 4C). Together, these results indicate that overexpressed GPR158 drives TBM-1 cell proliferation.

### Overexpression of GPR158 protein

**Figure 5:**
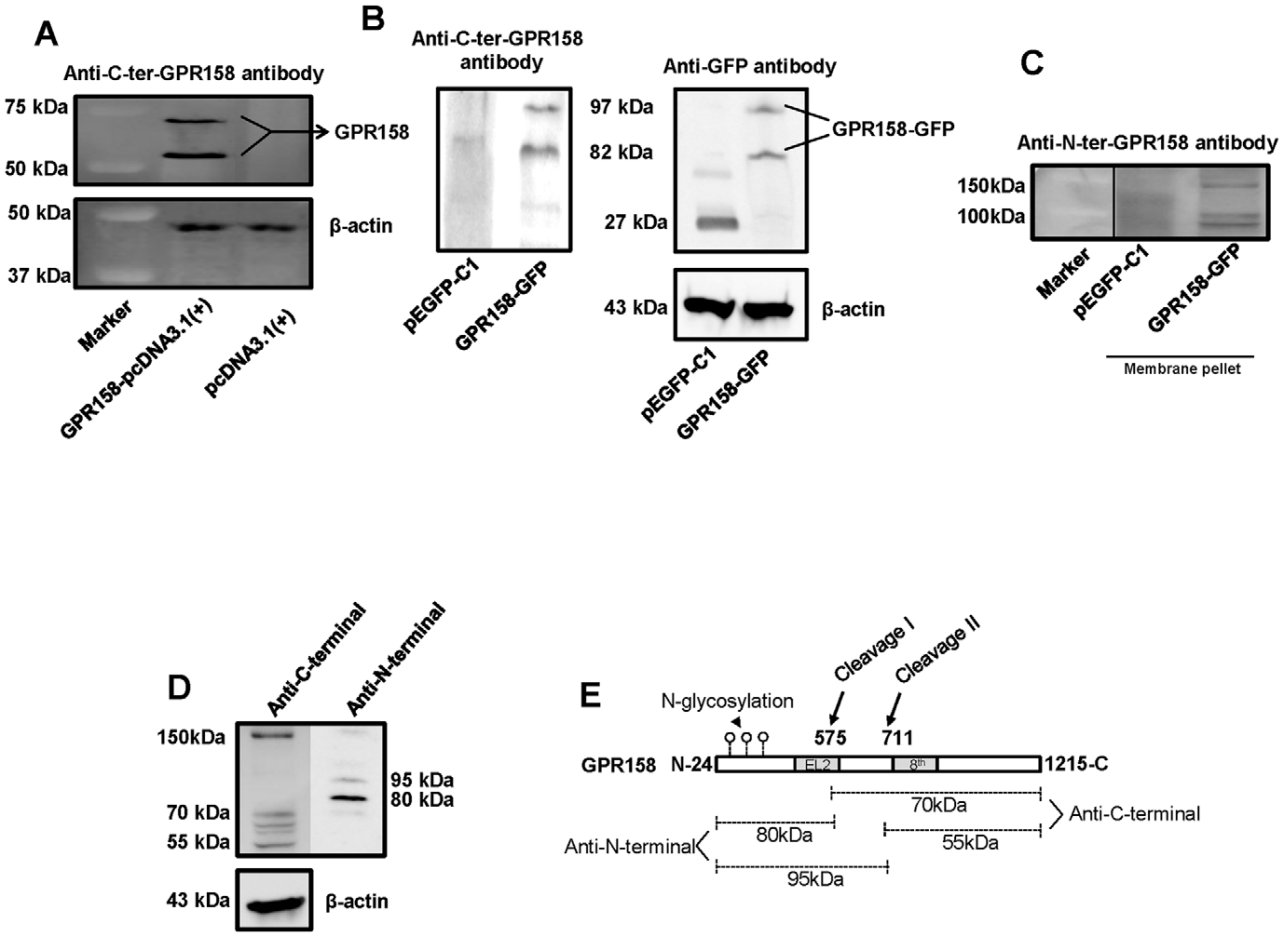
Overexpression of GPR158 protein. TBM-1 cells were transfected at 70–80% confluence using Lipofectamine LTX reagent with either indicated GPR158 expression plasmids or vector alone (**A, B and C**). Western blotting for the detection of GPR158 in whole cell lysates isolated from cells transfected with indicated plasmids using anti-C-terminal GPR158 antibody (**A and B**) and anti-GFP antibody (**B**). Total of 50 µg protein was subjected to SDS-PAGE analysis after boiling with 2-ME as indicated in Materials and Methods. The same membrane was striped and reprobed for β-actin for loading control. (**C**) The membrane pellet following cytosolic extract isolation from cells transfected with indicated plasmids was boiled in SDS-PAGE loading dye and loaded onto the gel. The western blotting was performed with anti-N-terminus GPR158 antibody. (**D**) Normal prostate tissue lysates from mice were isolated according to the procedure described in Materials and Methods. The total lysates (30 µg) was subjected to western blotting using either anti-C-terminal GPR158 or anti-N-terminal GPR158 antibodies. The results are representative of three different transfection experiments. ***P<.001; **P<.01; *P<.05; ns, P>.05. (**E**) The illustration of the location of two cleavage sites and corresponding sizes of the fragments, which are detected using anti-C- and anti-N-terminal GPR158 antibodies. Arrows indicated cleavage sites, I and II, and arrowhead indicate N-glycosylation in N-terminal extracellular domain.

As noted in the previous section, we determined GPR158 protein levels in our overexpression experiments by western blotting. These blots provided the opportunity to evaluate the status of the protein form, as well as to verify the specificity of our GPR158 antibodies. Fig. 5 shows results obtained using TBM-1 lysates, which were analyzed under reducing conditions from cells overexpressing GPR158 at 3 days post-transfection with either the pcDNA 3.1(+) construct or the GFP fusion construct.

GPR158 C-terminal antibody identified two separate bands of approximately 70- and 55-kDa in cytosolic lysates from cells transfected with the GPR158-pcDNA 3.1(+) construct (Fig. 5A). These bands were not detected in cells transfected with control vector, indicating their specificity (Fig. 5A). The presence of two bands, and their sizes, suggests that intracellular proteolytic cleavage occurs at two different sites in the overexpressed GPR158 protein: the EL2 loop and within sequences just proximal to the 8^th^ helix in the C-terminal cytoplasmic tail. The 150-kDa band corresponding to full-length GPR158 was not detected in cell lysates across many repeated transfection experiments, indicating that all of the overexpressed GPR158 protein is rapidly cleaved. In cytosolic lysates from cells overexpressing the GPR158-GFP fusion protein, two specific bands were also seen, but of 97- and 82 kDa (Fig 5B). These larger sizes are consistent with fusion of the 27-kDa GFP protein to the GPR158 C-terminus. Anti-GFP antibody detected fragments of identical size in the lysates from cells transfected with GPR158-GFP fusion construct, but detected only the 27-kDa GFP protein in cells transfected with control vector (Fig. 5B). The membrane pellet remaining after preparation of the cytosolic lysates was also evaluated by western blotting (Fig, 5C). The GPR158 N-terminal antibody detected a faint 150-kDa band corresponding to the full-length GPR158, but also detected two lower molecular weight bands of approximately 95- and 80-kDa corresponding to two N-terminal fragments. These sizes are consistent with the results with the C-terminal antibody, confirming the cleavage at two sites as described above.

In order to confirm that the proteolytic status of GPR158 cleavage is not specific to cultured TBM-1 cells, we evaluated GPR158 protein in normal prostate tissue from mice by western blotting under reducing conditions (Fig. 5D). The C-terminal GPR158 antibody detected three different bands corresponding to the full-length GPR158 (150-kDa) and two fragments with approximate sizes of 70- and 55-kDa. Similarly, the N-terminal GPR158 antibody detected the 150-kDa full-length GPR158 and bands of 95- and 80-kDa. This was as seen in TBM-1 cells overexpressing GPR158. Together, the cleaved fragments identified by both antibodies further confirm that the cleavage in GPR158 in normal prostate also occurs at the same location. The two cleavage sites and the resulting fragments sizes identified using both anti-C- and anti-N-terminal GPR158 antibodies are depicted in schematics (Fig. 5E). Together, these results show that overexpressed GPR158 in TBM-1 cells and endogenous GPR158 in mouse prostate are possibly subjected to proteolytic cleavage.

### Nuclear trafficking of GPR158

**Figure 6:**
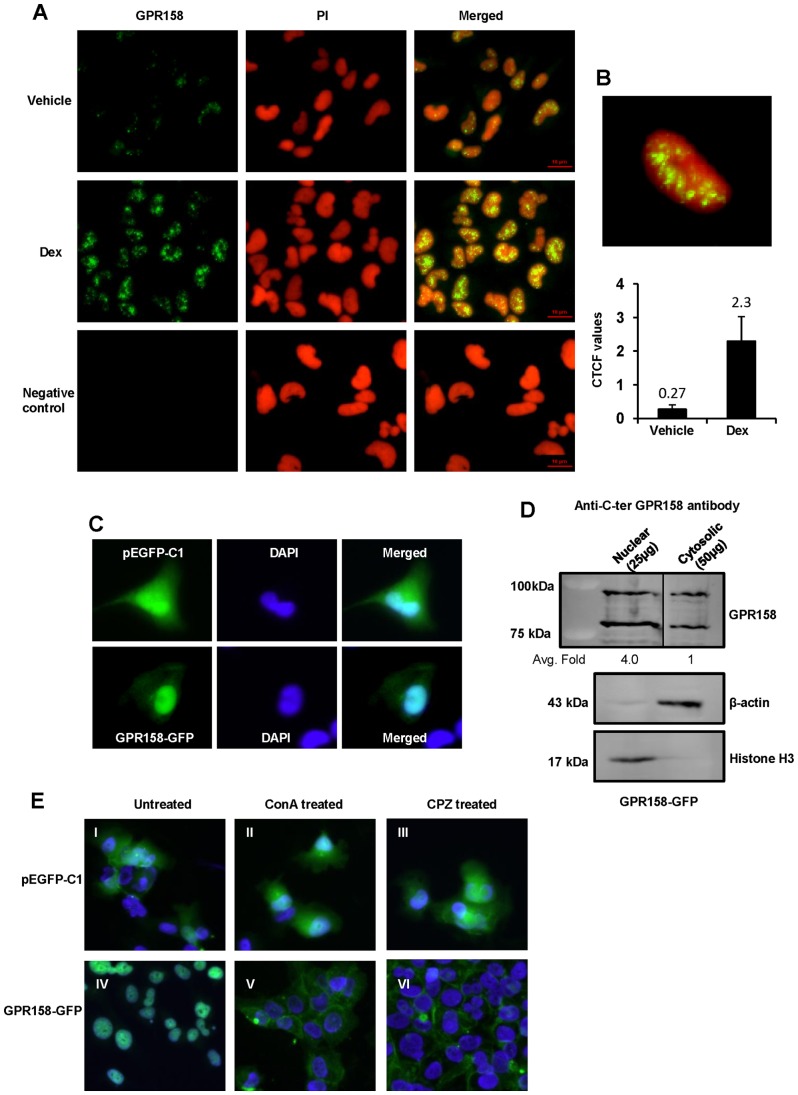
Nuclear trafficking of GPR158. (**A**) Indirect immunofluorescence microscopy was carried out using anti-C-terminal GPR158 antibody in vehicle treated (top panel) or Dex treated (middle panel) TBM-1 cells. The untreated cells labeled only with secondary and not with primary anti-GPR158 antibody, as negative control are indicated in bottom panel. Cell nucleus was labeled with propidium iodide (PI). All images were captured using Nikon Eclipse Ti-E fluorescence microscope at fixed acquisition parameters. Yellow indicates the colocalization of GPR158 with nuclear stain in merged images. (**B, top panel**) The image represents the *z*-stack projection of multiple confocal microscopy sections from the basal to the apical cell side, indicating the co-localization of GPR158 (green) with nuclear stain, PI (red). (**B, bottom panel**) The corrected total cell fluorescence (CTCF) from 50 individual cells in either vehicle or Dex treated sample was measured using NIH Image J software and normalized with negative control values. The bar diagram represent mean ± SEM of three independent experiments. The statistical analysis was carried out using unpaired Student's t tests (P<0.05). (**C**) The images were acquired using fluorescence microscopy of cells transfected with either vector or GPR158-GFP fusion plasmid. The representative image of single cell for both panels is shown from three independent transfection experiments. Post-transfection, the cells were fixed with 4% PFA, washed with PBS and the slides were mounted using VECTASHIELD with DAPI. The images were acquired on Nikon Eclipse Ti-E fluorescence microscope. (**D**) The nuclear extracts (25 µg) and cytosolic extracts (50 µg) isolated from cells overexpressing GPR158-GFP fusion protein were subjected to SDS-PAGE and western blotting to detect GPR158 using anti-C-terminal GPR158 antibody (dilution: 1∶1000), as described in Materials and Methods. The purity of nuclear and cytosolic extracts in immunoblotting was confirmed using anti-beta actin and anti-histone H3 antibodies. Quantification of both fragments of GPR158 protein band intensities was measured by NIH Image J and expressed in terms of fold levels relative to levels in cytosolic extracts considered as 1.0. The statistical analysis was carried out using unpaired Student's t tests (P<0.05). The average fold and P values obtained from three different experiments are indicated in the figure. The vertical line indicates repositioned gel lanes. ***P<.001; **P<.01, *P<.05; ns, P>.05. (**E**) TBM-1 cells were either transfected with GFP vector alone (I, II and III) or GPR158-GFP plasmid (IV, V and VI). 3 days after transfection, the cells were either incubated in culture media (I and IV) or media containing ConA (II and V) (0.25 mg/mL) for 8 hrs or CPZ (III and VI) (10 µM) for 3 hrs. Following treatment, the cells were washed with PBS and fixed with 4% PFA for 20 mins at RT. The cells were washed again and mounted with VECTASHIELD containing DAPI and images were acquired with a Nikon Eclipse Ti-E fluorescence microscope. The images represent two independent experiments.

GPCRs are typically expressed on the plasma membrane, where they are available to bind their extracellular ligand. Immunohistochemical staining data deposited to the HPA database [9], utilizing the same C-terminal GPR158 antibody that we used in the studies described above, demonstrates both cytoplasmic/membranous and nuclear localization for GPR158 in various tissues. We carried out indirect immunofluorescence experiments with this antibody to determine the subcellular localization of GPR158 in TBM-1 cells. Representative results are shown in Fig. 6. Treatment of TBM-1 cells with Dex led to a significant increase in GPR158 expression (Fig. 6A, middle panel), as shown by much stronger fluorescence intensity detected with the C-terminal GPR158 antibody, in comparison with vehicle treated cells (Fig 6A, top panel). Untreated cells labeled only with secondary antibody as a negative control, showed only weak fluorescence (Fig. 6A, bottom panel). The cells were counterstained with propidium iodide to identify nuclei; the merged immunofluorescence images suggested that GPR158 was localized entirely to the nucleus of the cell. We confirmed the nuclear localization of GPR158 by examining confocal z-stack sections, which showed clear co-localization of GPR158 (green) in punctate bodies within the nucleus stained with propidium iodide (red) as shown in a representative z-stack image (Fig. 6B, top panel). The increased GPR158 expression with Dex stimulation was quantified using corrected total cell fluorescence (CTCF) through Image J software of randomly selected 50 individual cells in each sample, according to the software's instructions. The results in Fig. 6B, bottom panel, showed approximately 8.5-fold increase in fluorescence intensity of GPR158 staining in Dex-treated cells, compared with vehicle-treated cells.

We next examined the intracellular localization of GPR158-GFP in transiently transfected TBM-1 cells. Representative results are shown in Fig. 6, C and D. In comparison to widespread GFP expression in cells transfected with control vector, GPR158-GFP showed preferential nuclear localization (Fig. 6C). In order to further demonstrate the nuclear localization of GPR158 in cells transfected with GPR158-GFP plasmid, we performed a western blot with nuclear extracts isolated using the hypotonic salt method. The results showed the presence of two cleaved fragments of GPR158 in both nuclear and cytosolic extracts of TBM-1 cells overexpressing GPR158 using anti-C-terminus GPR158 antibody (Fig. 6D). Additionally, histone H3, a highly specific nuclear marker is detected only in the nuclear fractions while β-actin was found only in the cytosolic fraction of TBM-1 cells overexpressing GPR158-GFP, indicating efficient separation and absence of any contamination of the nuclear and cytoplasmic extracts (Fig. 6D). Furthermore, quantification of western blot densitometry using the Image J processing program (NIH) identified a 4-fold enrichment of GPR158 protein in the nuclear fraction compared to the cytosolic fraction.

In the standard GPCR paradigm, extracellular ligand binding at the cell surface leads to receptor activation, followed by rapid inactivation by internalization into the cytoplasm via endocytic vesicles [23]. To learn whether GPR158 traffics to the plasma membrane in TBM-1 cells, we treated GPR158-GFP over-expressing cells with two different inhibitors of endocytosis: ConA [24] and CPZ [25]. Representative results are shown in Fig. 6E. ConA treatment had no effect on GFP distribution in cells overexpressing vector alone (compare Fig. 6E, I and II). In contrast, ConA treatment of GPR158-GFP transfected cells clearly shifted localization of GFP fluorescence to the plasma membrane (compare Fig. 6E, IV and V). Moreover, treatment of GPR158-GFP expressing cells with CPZ further confirmed GPR158 localization to the plasma membrane (compare Fig. 6E, IV and VI). In comparison, CPZ treatment of vector only transfected cells did not show any change in GFP localization (compare Fig. 6E, I and III). These results demonstrate that, despite its localization to the nucleus in TBM-1 cells, GPR158 displays the typical behavior of GPCR clan members by first trafficking to the plasma membrane.

### Role of the bipartite NLS in nuclear localization and cell proliferation

As noted above, conceptually translated GPR158 protein contains a bipartite NLS in the 8^th^ helix. A previous *in silico* analysis of over 200 family A GPCR sequences revealed 17 family members with a clearly recognizable NLS motif in the 8^th^ helix. Functional studies showed that this motif mediates ligand-independent nuclear translocation of family A GPCRs when present specifically at this site [26]. We performed a similar analysis of all family C GPCRs (Table 2). The PSIPRED program revealed that 15 of the 22 genes encoding family C GPCRs contain an 8^th^ helix. PredictNLS revealed that 8 of these genes contain an NLS motif in the 8^th^ helix, including GPR158. The NucPred server [19] contains a set of programs that analyze patterns in eukaryotic protein sequences and predict if a protein spends time in the nucleus. Sequences which score ≥ 0.8 with NucPred, and which are predicted by PredictNLS to contain an NLS, have been shown to be 93% accurate. A likelihood score is derived from the programs for each input sequence and each residue position. Table 2 shows our determination of the NucPred score for all 22 members of family C GPCRs. Interestingly, the three orphans of the GABA branch had the highest scores by far, each close to or above 0.8. GPR158 had the top score of 0.96, indicating a very high likelihood that the protein would localize to the nucleus (a score of 1.0 is perfect).

**Table 2:** NucPred Analysis of the 22 Members of GPCR Family C.

Name	8^th^ helix	NLS in 8^th^ helix	NLS elsewhere	NucPred Score
mGlu1	+	+	+	0.46
mGlu2	−	−	+	0.08
mGlu3	+	−	+	0.11
mGlu4	+	+	−	0.55
mGlu5	+	−	−	0.45
mGlu6	+	+	−	0.36
mGlu7	+	+	−	0.58
mGlu8	+	+	−	0.48
GPRC6A	+	−	−	0.3
CaR	+	−	+	0.61
T1R1	+	−	−	0.06
T1R2	−	−	−	0.21
T1R3	+	−	+	0.15
GABAB2	−	−	+	0.43
GABAB1	+	+	+	0.33
GPR156	+	−	+	0.78
GPR158	+	+	+	0.96
GPR179	+	+	+	0.85
GPRC5D	−	−	−	0.09
GPRC5A	−	−	+	0.11
GPRC5B	−	−	+	0.02
GPRC5C	−	−	+	0.21

The PSIPRED program [20] predicted the presence of 8^th^ helix and the NucPred score represents the threshold score for the fraction of proteins, correctly predicted as nuclear (specificity) versus the fraction of true nuclear proteins predicted (sensitivity) at or below given score [19].

**Figure 7:**
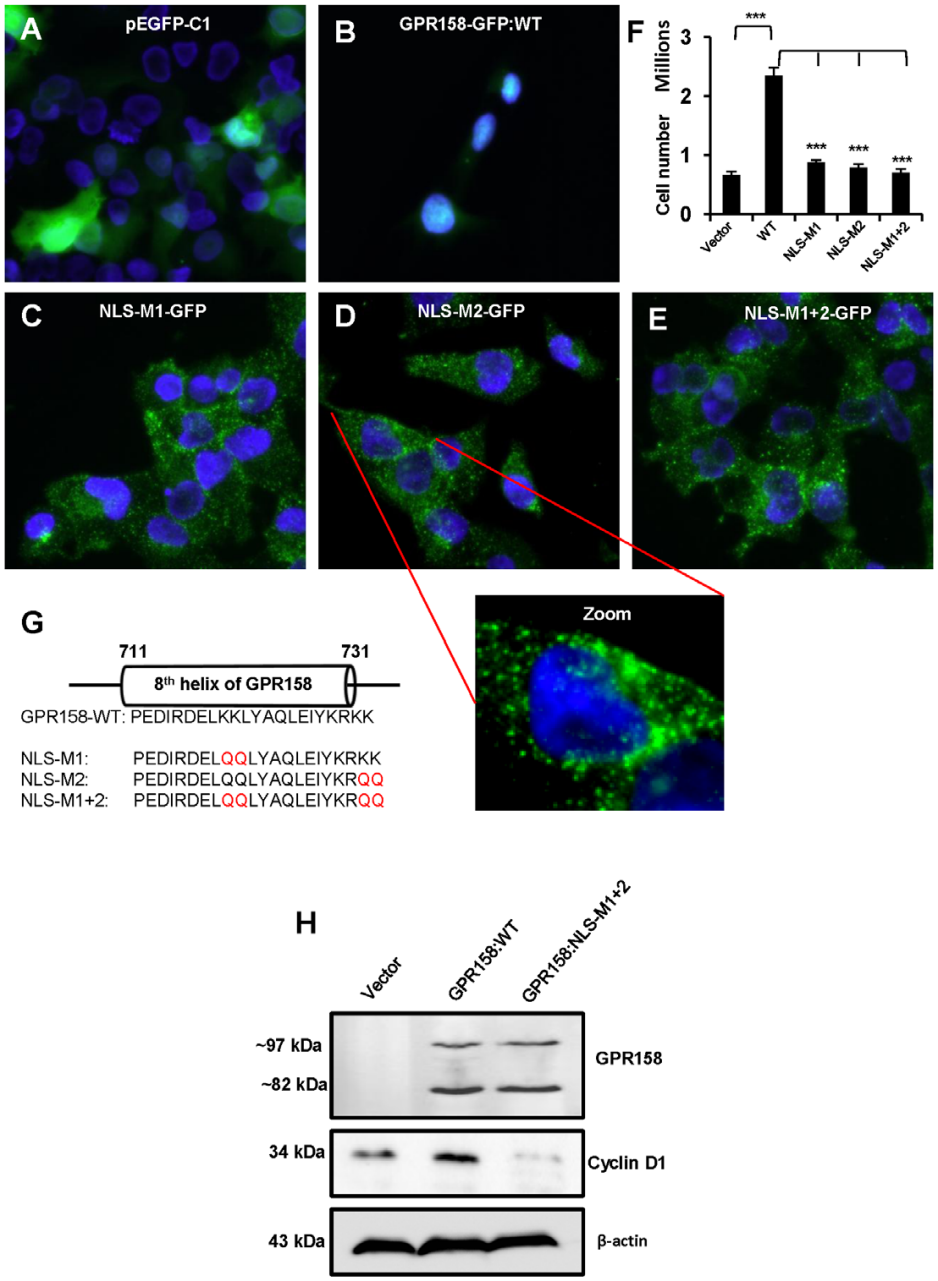
Role of the bipartite NLS in nuclear localization and cell proliferation. (**A, B, C, D and E**) The fluorescent images were captured of cells transfected with either vector (**A**) or GPR158-GFP plasmid (**B**) or NLS-M1-GFP plasmid (**C**) or NLS-M2-GFP plasmid (**D**) or NLS-M1+2-GFP plasmid (**E**). The representative images from two independent transfection experiments are shown. 3 days post-transfection, the cells were fixed with 4% PFA, washed with PBS and the slides were mounted using VECTASHIELD with DAPI and viewed using Nikon Eclipse Ti-E fluorescence microscope. The merged images show GPR158-GFP as green and nuclear stain DAPI as blue. The zoom panel indicates the magnified image of an indicated cell. (**F**) After 3 days of transfection, the cells transfected with above indicated plasmids were trypsinized and counted using trypan blue dye in a hemocytometer chamber. The data represent mean ± SEM of two independent experiments. (**G**) Schematics showing amino acid sequence corresponding to the bipartite NLS, a part of 8^th^ helix of GPR158. The mutated amino acids are shown in color red in indicated NLS mutant constructs. (**H**) TBM-1 cells were transfected with either GPR158 wild type or NLS-M1+2 or GFP vector alone plasmid using Lipofectamine LTX reagent. After 3 days of transfection, the cell lysates were prepared using RIPA buffer and the western blotting for the detection of GPR158 and cyclin D1 was performed using appropriate antibodies. The same membrane was striped and reprobed for β-actin for loading control. The data represent two independent experiments performed in triplicate.

To test experimentally the role of the GPR158 bipartite NLS in nuclear localization, we generated three different NLS mutations in our GPR158-GFP construct, altering the proximal NLS (M1), the distal NLS (M2), or both (M1+M2). The mutated amino acids and their locations with respect to the 8^th^ helix are shown in the schematics (Fig. 7G). Each of these three mutated constructs was then individually overexpressed in TBM-1 cells. Representative results are shown in Fig, 7, A-E. As expected, GFP fluorescence was broadly distributed in cells transfected with vector alone (Fig. 7A), but was predominantly localized in the cell nucleus in cells transfected with GPR158-GFP (Fig. 7B). In contrast, GFP fluorescence was strikingly localized in punctuate vesicles distributed in the cytosol in cells overexpressing NLS-M1, NLS-M2 or NLS-M1+2 mutants, with a concentration of vesicles at, or close to, the plasma membrane (Fig. 7, C, D and E). The magnified image of a single cell representative of staining pattern obtained with all three NLS mutants clearly shows the vesicular localization of GPR158, both in cytosol and underneath the plasma membrane with a remarkable loss in nuclear staining as compared to wild type GPR158 (Panel marked as Zoom). These results demonstrate the requirement of the bipartite NLS in nuclear localization of GPR158. They are consistent further with a mechanism whereby newly synthesized GPR158 first traffics to the plasma membrane, where it is then rapidly internalized in small endocytic vesicles for translocation to the nucleus.

Since NLS mutants failed to translocate to the nucleus, we performed experiments to determine whether there was any change in cell proliferation in cells overexpressing these mutants. Interestingly, all three NLS mutants failed to increase TBM-1 cell proliferation in the absence of their nuclear translocation, while wild-type GPR158-GFP overexpression led to a 3.5-fold increase in cell proliferation (Fig. 7F). To understand more about GPR158 effects on cell proliferation, we evaluated the levels of cyclin D1, a critical player in the progression of cell cycle from G1 to the S phase [27] in cells overexpressing GPR158 wild type or NLS-M1+2 mutant. The results in Fig. 7H showed significantly increased levels of cyclin D1 protein in wild type GPR158 overexpressing cells, in comparison to vector transfected cells. However, the increased cyclin D1 expression was completely abrogated in cells overexpressing GPR158:NLS-M1+2 mutant. The top panel, Fig. 7H confirmed the overexpression of GPR158 protein in cells overexpressing either wild type or NLS-M1+2 mutant. Together, these results demonstrate the requirement of nuclear localization for GPR158 to promote cell proliferation, and indicate that nuclear translocated GPR158 promotes cell proliferation by modulation of cyclin D1 expression.

### GPR158 promotes TBM barrier function

**Figure 8:**
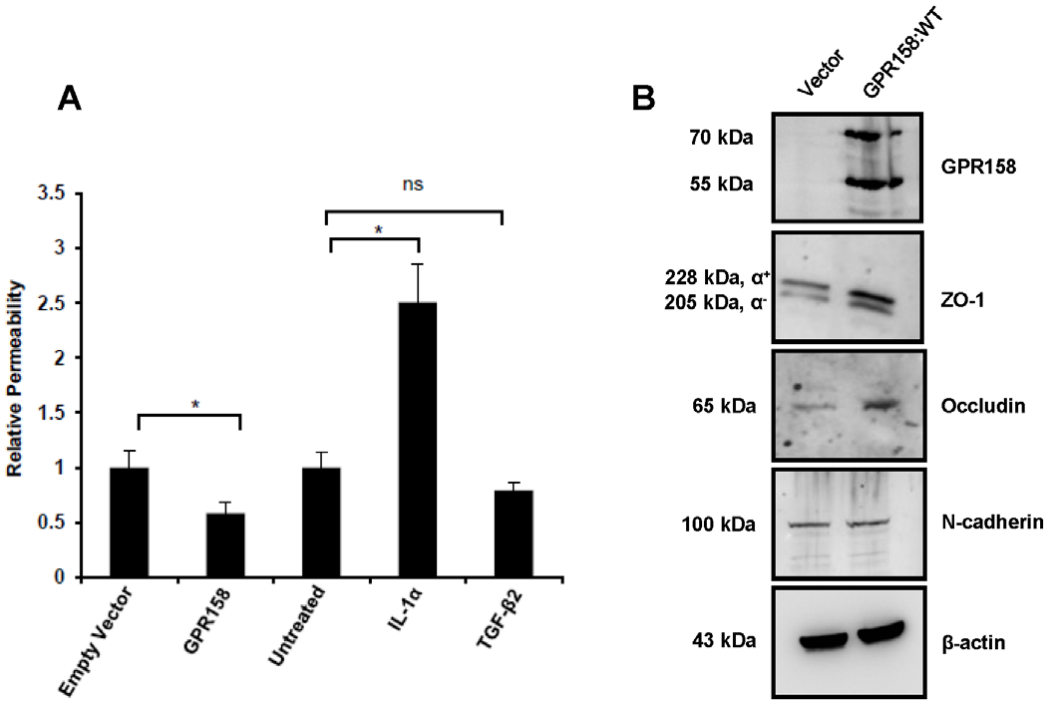
Overexpression of GPR158 significantly reduced TBM monolayer permeability. (**A**) IVP assay was carried out as described in materials and methods. The data represent mean ± SEM of 3 independent experiments and p-values were calculated by student t test. *p<0.05 and ns, p>0.05. (**B**) TBM-1 cells were transfected with either pcDNA3.1(+) vector alone or GPR158-pcDNA3.1(+) expression plasmid using Lipofectamine LTX reagent. The cell lysates were prepared using RIPA buffer at 3-days post-transfection and the western blotting for the detection of GPR158, ZO-1, occludin and N-cadherin was performed using appropriate antibodies. The same membrane was striped and reprobed for β-actin for loading control. The data represent two independent transfection experiments performed in triplicate.

Modulation of paracellular permeability of the barrier is thought to be a regulator of aqueous outflow facility [28]. In a primary TBM or Schlemm's canal cell culture monolayer model, barrier function is decreased by IL-1 alpha and increased by TGF-beta2 [28]. We evaluated the effects of GPR158 overexpression on TBM barrier function by measuring the paracellular permeability of FITC-dextran across a primary TBM cell monolayer grown on permeable filters using IVP assay. Fig. 8A shows that TBM cells overexpressing GPR158 were significantly less permeable to FITC-dextran than empty-vector transfected cells. In control experiments, TBM cell monolayers treated with IL-1alpha (10 ng/ml) or TGF-beta2 (10 ng/ml) for 24 hrs prior to assessing permeability, showed significantly increased and moderately reduced permeability, respectively in comparison to untreated cells, as previously reported [28]. Moreover, similar to GPR158 overexpressed cells, cells treated with Dex (100 nM) for 24 hrs also showed significantly reduced permeability as compared to cells treated with vehicle control, as previously reported [29] (data not shown).

Tight junctions (TJ) are key regulators of barrier function in the outflow pathways [29] and other tissues [30]–[31]. We evaluated TJ formation biochemically by western blotting for the TJ specific proteins, ZO-1 and occludin. The results in Fig. 8B demonstrated a clear increase (3-fold) in the levels of both ZO-1 and occludin in TBM cells overexpressing GPR158 compared with vector transfected cells. In contrast, levels of N-cadherin, a cell adhesion molecule that is not part of the TJ, remained unchanged in GPR158 overexpressing cells. Together, these results indicate that increased GPR158 expression leads to elevated levels of proteins involved in TJ formation, consistent with reduced permeability of the TBM cell monolayer.

## Discussion

The present study characterizes GPCR Family C orphan GPR158, a relatively unstudied GPCR that we identified in a pilot pharmacogenomic study on GC-induced OH. We report that GPR158 is GC-inducible through transcriptional mechanisms in TBM cells derived from the eye's aqueous outflow pathway. In addition, we find an unusual nuclear localization for GPR158, demonstrate intracellular trafficking pathways by which this occurs, and show that it has functional consequences for control of cell proliferation. Like treatment with GCs, overexpression of GPR158 also enhances the barrier function of a primary TBM cell culture monolayer. Regulated paracellular permeability of the TBM barrier is one factor controlling aqueous outflow facility *in vivo*. Since GCs stimulate GPR158 expression, the results are consistent with a role for excessive GPR158 expression in GC-induced OH.

GCs are used therapeutically in a wide variety of diseases, however they can have significant side effects in susceptible individuals. Administration of GCs in the eye causes OH in a subset of patients [32], [33]. Histological and molecular studies suggest that steroid-induced OH and OH leading to POAG are mechanistically similar, both caused by a deficiency in aqueous outflow facility through the TBM and Schlemm's canal [28]. TGF-beta superfamily signal transduction has been linked with pathology [34], while IL-1 is compensatory and protective [35]. Interestingly, MYOC, the first glaucoma gene discovered, encodes a GC-induced protein called myocilin. However, later studies revealed no evidence for a link between MYOC mutations and GC-induced OH [22].

GCs bind to and activate intracellular glucocorticoid receptors (GRs) that move to the nucleus and bind DNA as transcription factors, activating or inhibiting expression of a set of genes. We found that TBM cells express only very low levels of GPR158 mRNA and protein, but treatment with GCs stimulates expression to easily detectable levels. TBM-1 cells displayed late GC induction for GPR158 expression, similar to MYOC [22] [36]. As we show here for GPR158, the 5-kilobase region upstream of the MYOC transcriptional start site also contains several GREs. Expression of MYOC progressively increases from barely detectable levels to greater than 2% of total cellular mRNA over 10 days exposure of TBM cells to Dex. This suggests both primary and secondary mechanisms at play in the complete response to GCs [36], [37], as has been reported in the regulation of hepatic alpha 2u-globulin gene expression [38].

Immunohistochemical staining data deposited to the HPA database [9] demonstrates both cytoplasmic/membranous and nuclear localization for GPR158 in the same tissue. In the recently published study on retinal GPR158, transient overexpression of GPR158 in HEK293T/17 along with RGS7 resulted in plasma membrane localization [11]. While we clearly demonstrated nuclear localization of both endogenous and overexpressed GPR158, further experiments showed that GPR158 is shifted to the plasma membrane by treatment with ConA or CPZ. ConA inhibits endocytosis of many receptors, probably by its ability to irreversibly cross-link glycosylated proteins [24] while CPZ is a more specific inhibitor of clathrin-mediated endocytosis [25]. Thus our results are consistent with the notion that GPR158 traffics to the plasma membrane, as is typical for GPCRs, prior to endocytosis and subcellular localization to other compartments. It will be interesting to learn more about the circumstances causing retention at the plasma membrane, as it seems likely this has functional significance.

Protein translocation into the nucleus is regulated by importins, which bind to the NLS motif [39]. Beta-arrestin 1 may also participate in this process based on its well-established role in receptor internalization and nuclear translocation [40]. It was previously shown that when a NLS is specifically present in the 8^th^ helix, it mediates ligand-independent nuclear translocation [26]. In this study, we show that an 8^th^ helix is also present in 15 of the 22 members of GPCR family C, including GPR158, which contained a bipartite NLS. In our studies with overexpressed GPR158-GFP, we detected two cleaved fragments containing the C-terminal GFP fusion in the cell nucleus, suggesting that proteolytic cleavage could be a part of the mechanism for GPR158 nuclear translocation. This behavior is similar to that seen for a Drosophila GPCR of a different family, DFrizzled2 (dfz2), whose c-terminal cytoplasmic tail is cleaved coincident with binding of its WNT ligand, followed by rapid translocation to the nucleus [41].

GPCRs signal through G proteins in response to extracellular ligand binding. An extracellular ligand for orphan GPR158 has yet to be identified, and was not explored in this study. Since no agonist was added to the cell culture medium, we expected GPR158-GFP to be stranded on the plasma membrane when the NLS was mutated. Instead, it was detected within vesicles clustered beneath the plasma membrane and throughout the cytoplasm. These results suggest that GPR158 may be constitutively active like some other GPCRs [42]. Alternatively, GPR158 may be activated by an unknown ligand in the cell culture medium, perhaps secreted by the cells themselves. Intracellular allosteric modulators may also activate GPR158 similar to metabotropic glutamate receptors [43]. On the other hand, GPR158 may not function through G proteins at all. Orlandi et al. showed that plasma membrane GPR158 acts as an anchor for RGS7 complexes [11], thus may modulate the signaling of other GPCRs. GPR158 lacks the ligand binding VFT domain, but it has conserved amino acids involved in G protein binding [43]. Further studies will be necessary to determine the importance of these domains.

GC treatment of primary TBM cell cultures causes reorganization of actin stress fibers into cross-linked actin networks (CLANs) [44] and a marked enlargement of TBM nuclei and DNA content has also been reported [29]. We found that GCs enhanced cell proliferation in an immortalized TBM-1 cell line. Our observations are in accordance with a previously published study using another immortalized human TBM cell line [45]. The authors of this study suggested that a disease correlate might be the proliferation of cells in the inner wall of Schlemm's canal in congenital glaucoma, leading to deposition of fibrous connective tissue [46].

In fact, one of the most significant findings of the present study was the demonstration of a functional role for nuclear localized GPR158. Many overexpressed GPCRs stimulate cell proliferation through generalized activation of ERK signaling and/or other pathways. However, we showed that knockdown of endogenous GPR158 or overexpression of NLS mutants clearly inhibits cell proliferation, supporting a more specific role for nuclear localized GPR158 in stimulating cell proliferation. Accumulating evidence indicates that GPCRs can elicit G-protein signaling at other subcellular sites including the nucleus [47], [48]. However, the precise mechanism by which nuclear GPR158 regulates cell proliferation remains to be established. Our results support the involvement of cyclin D1, an important regulator of cell cycle progression from G1 to S phase, in linking nuclear function of GPR158 to cell proliferation. In addition, we identified several phosphorylation sites in the cytoplasmic tail of GPR158 for protein kinases involved in cell cycle progression. UniProt database identified binding of transcription factors c-Myc and Pitx2 to specific sequences located just distal to the 8^th^ helix of GPR158. Since, GPR158 localizes to the nucleus, the possibility of a functional role for these protein-protein interactions exists. Myc expression is activated upon various mitogenic signals and controls cell proliferation [49]. The Pitx2 protein regulates the development of the eye, teeth and abdominal organs [50]. Mutations in this gene are associated with developmental disorders of the eye's anterior segment that result in OH [51], including Axenfeld-Rieger syndrome, iridogoniodysgenesis syndrome, and Peter's anomaly [50]. Like Myc, Pitx2 also directly activates transcription of genes that regulate the cell cycle [52]. PITX2 was also recently shown to modulate response to oxidative stress in TBM cells, a condition associated with OH leading to POAG [53].

Our results on the role of GPR158 in barrier function of a TBM monolayer are strikingly similar to findings by Underwood et al., 1999, which identified reduced permeability of TBM cells when treated with GC, due to increased levels of ZO-1, marker for TJs formation [29]. Also, our results are similar to an increasing body of evidence that supports the key role for ZO-1-occludin complex and ZO-1's isoforms in the fluid flow regulation across many different cellular barriers [30], [31]. In fact, our results of higher content of ZO-1α^+^ isoform in GPR158 overexpressing cells support the reported relationship between ZO-1 isoforms and flow resistance, where ZO-1α^+^ confers greater resistance than ZO-1α^−^ [54]. The *in vitro* TBM monolayer permeability assay as a model to study outflow resistance has been widely used and supported by experiments conducted with several medications that are known to alter aqueous humor outflow *in vivo*, also demonstrate similar regulation in cultured cells [55]. [29]. Based on our results of increased expression of endogenous GPR158 by GC and capacity of GPR158 to modulate permeability in cell culture model, it is reasonable to postulate that GPR158 may mediate some of the physiological functions of GC. The studies are currently underway to delineate the direct and indirect functions of GPR158 in TBM cells treated with GC. Further studies are needed to verify the role of GPR158 in normal aqueous outflow or OH *in vivo*.

The monolayer permeability assay used in this study provided us an opportunity to assess the role of GPR158 in barrier function similar to that of the vasculature or Schlemm's canal [14] in the aqueous outflow pathways. The results must be interpreted cautiously because TBM cells are not identical to Schlemm's canal cells, and even Schlemm's canal cells do not lay down a continuous basement layer in the actual human TBM. In addition, the monolayer permeability assay is a simplified model that does not mimic several physiological aspects of the aqueous outflow pathway, such as anatomical existence of TBM as three differentiated layers, the presence of extracellular matrix, its autonomic and sensory innervation and its contractility. Therefore, further studies using models that mimic these physiological characteristics of the aqueous outflow pathway, such as the anterior segment–perfused organ culture model and animal models are warranted for validating the role of GPR158 in OH. Overall, our findings represent promising initial steps in understanding the role of GPR158 in the outflow pathway and its possible link with GC-induced OH.

In summary, our findings identify for the first time, the nuclear localization of GPR158 through a functional bipartite NLS and the essential role for this translocation in control of cell proliferation. Our data provide direct evidence that excess GPR158 could contribute to reduced TBM cell monolayer permeability and therefore modulate aqueous outflow. It will be interesting to study the possible mechanisms whereby GPR158 traffics to the nucleus or is retained on the plasma membrane, and the functional implications. Pharmaceutical-assisted retention of GPR158 at the plasma membrane could be a novel strategy for inhibiting nuclear activities of GPR158 in these and other disease processes.

## Supporting Information

Figure S1 **GPR158 knockdown using a pool of three siRNAs.** PC-3 cells were transfected at 80% confluence using Lipofectamine LTX reagent with either a pool of three GPR158 siRNAs or control scrambled siRNA at indicated concentration. After 3 days of transfection, the cells were trypsinized, washed with PBS, lysed with RIPA buffer and cell lysates were subjected to western blotting using anti-C-terminal GPR158 antibody. β-actin was used as a loading control. Quantification of GPR158 protein band intensities was measured by NIH Image J, normalized by β-actin band intensities and expressed in terms of fold expression relative to levels in untransfected control cells arbitrarily set at 1.0. The average fold from three different experiments is indicated in the figure.
(TIFF)
