# Supplementary figures and images for: GPR158, an Orphan Member of G Protein-Coupled Receptor Family C: Glucocorticoid-Stimulated Expression and Novel Nuclear Role
Source: PLoS One. 2013 Feb 25;8(2):e57843. doi: 10.1371/journal.pone.0057843 (PMC3581496; doi:10.1371/journal.pone.0057843)

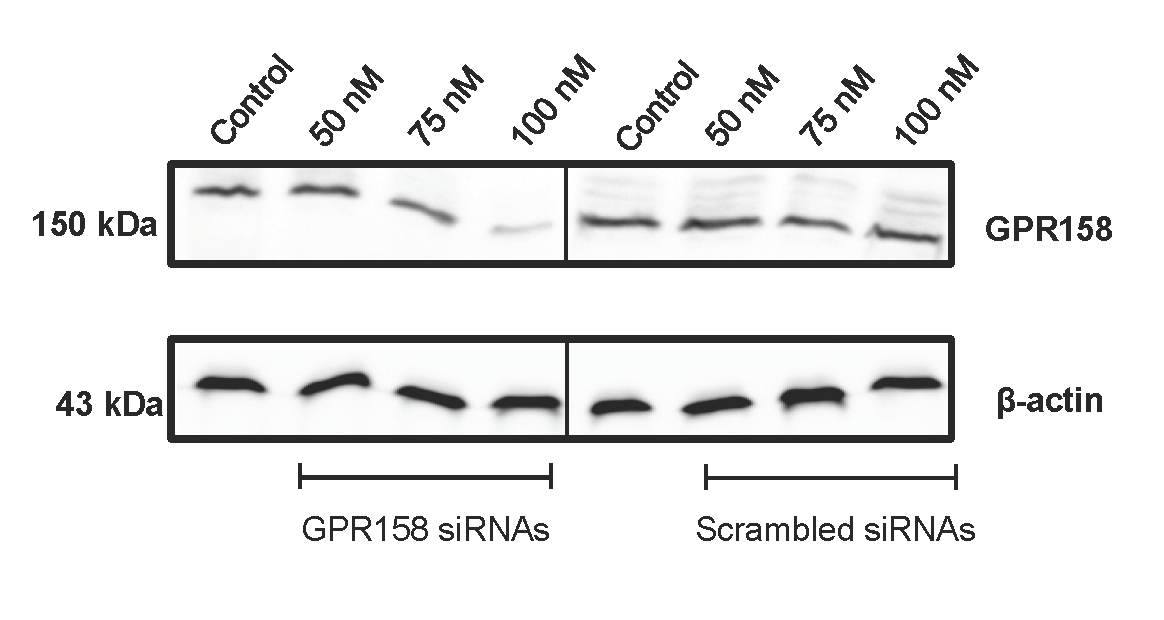

Supplement: Figure S1 — GPR158 knockdown using a pool of three siRNAs. PC-3 cells were transfected at 80% confluence using Lipofectamine LTX reagent with either a pool of three GPR158 siRNAs or control scrambled siRNA at indicated concentration. After 3 days of transfection, the cells were trypsinized, washed with PBS, lysed with RIPA buffer and cell lysates were subjected to western blotting using anti-C-terminal GPR158 antibody. β-actin was used as a loading control. Quantification of GPR158 protein band intensities was measured by NIH Image J, normalized by β-actin band intensities and expressed in terms of fold expression relative to levels in untransfected control cells arbitrarily set at 1.0. The average fold from three different experiments is indicated in the figure. (TIFF) [file pone.0057843.s001.tif]
